# Spore immobilized enzymes for the multi-step synthesis of cellobiose

**DOI:** 10.1186/s12934-026-02943-w

**Published:** 2026-02-03

**Authors:** Jan Benedict Spannenkrebs, Leesa Jane Klau, Marianna Karava, Finn Lillelund Aachmann, Johannes Kabisch

**Affiliations:** 1 Department of Biotechnology and Food Science, NTNU Norwegian University of Science and Technology, Trondheim, Norway; 2 SINTEF Industry, Forskningsveien 1, Oslo, Norway; 3 Austrian Center of Industrial Biotechnology, Petersgasse 14, Graz, Austria

**Keywords:** Sucrose phosphorylase, Cellobiose phosphorylase, Bacillus subtilis, Reaction cascade, Spore display, Glucose 1-phosphate, Immobilization, NMR

## Abstract

**Background:**

Cellobiose (4-*O*-β-D-Glucopyranosyl-D-glucopyranose) is an important disaccharide utilized, for example in food and cosmetics. It can be enzymatically synthesized involving two steps from sucrose and glucose, where first the sucrose undergoes phosphorolysis by sucrose phosphorylase, yielding glucose 1-phosphate and fructose. Glucose 1-phosphate is then combined with glucose into cellobiose, releasing phosphate in a reaction catalyzed by cellobiose phosphorylase. To better control and reuse the enzymes in the two main reaction steps, immobilization on *Bacillus subtilis* spores is a promising approach due to the ease of production and recyclability.

**Results:**

Here we describe the display of a sucrose phosphorylase and a cellobiose phosphorylase on *B. subtilis* spores through fusion with the crust protein CotY, to our knowledge marking the first use of multiple enzymes directly displayed on the spore surface during sporulation in a reaction cascade. While immobilization had no effect on thermostability, we demonstrate the recyclability of the individual spore variants over four reaction cycles at 45 °C with sucrose phosphorylase maintaining 35% of its initial activity and cellobiose phosphorylase maintaining 65%. Both spore variants were used together to catalyse a reaction cascade in a separated two-pot, as well as in a one-pot reaction. The one-pot reaction achieved a 90% yield with respect to the initially available 40 mM of glucose. The one-pot cascade maintained activity after being recycled five times over the course of 120 hours. Furthermore, we report on improving the reaction yield in the two-pot reaction from 60% to 80% by using calcium to precipitate excess phosphate.

**Conclusion:**

In this study we demonstrate that spores are a suitable immobilization platform for multistage reaction cascades. The spores displaying biocatalysts can be recovered and reused over multiple reaction cycles. The immobilization of glycosylic enzymes on spores enables cost-effective, scalable enzyme production on a temperature-resistant carrier that facilitates purification. The potential modularity of this approach adds to the adaptability of the system to different requirements in terms of substrate and product.

**Supplementary Information:**

The online version contains supplementary material available at 10.1186/s12934-026-02943-w.

## Background

 Cellobiose is a carbohydrate with diverse applications. The reducing sugar is composed of two β-1,4-linked glucose molecules, exhibits prebiotic properties, inhibits pathogenic microorganisms, and has an 80% reduced relative sweetness compared to sucrose [1]. It is used as a food additive [2, 3], in the formulation of pharmaceuticals [4] and has been investigated as a cryoprotectant [5].

**Fig. 1:**
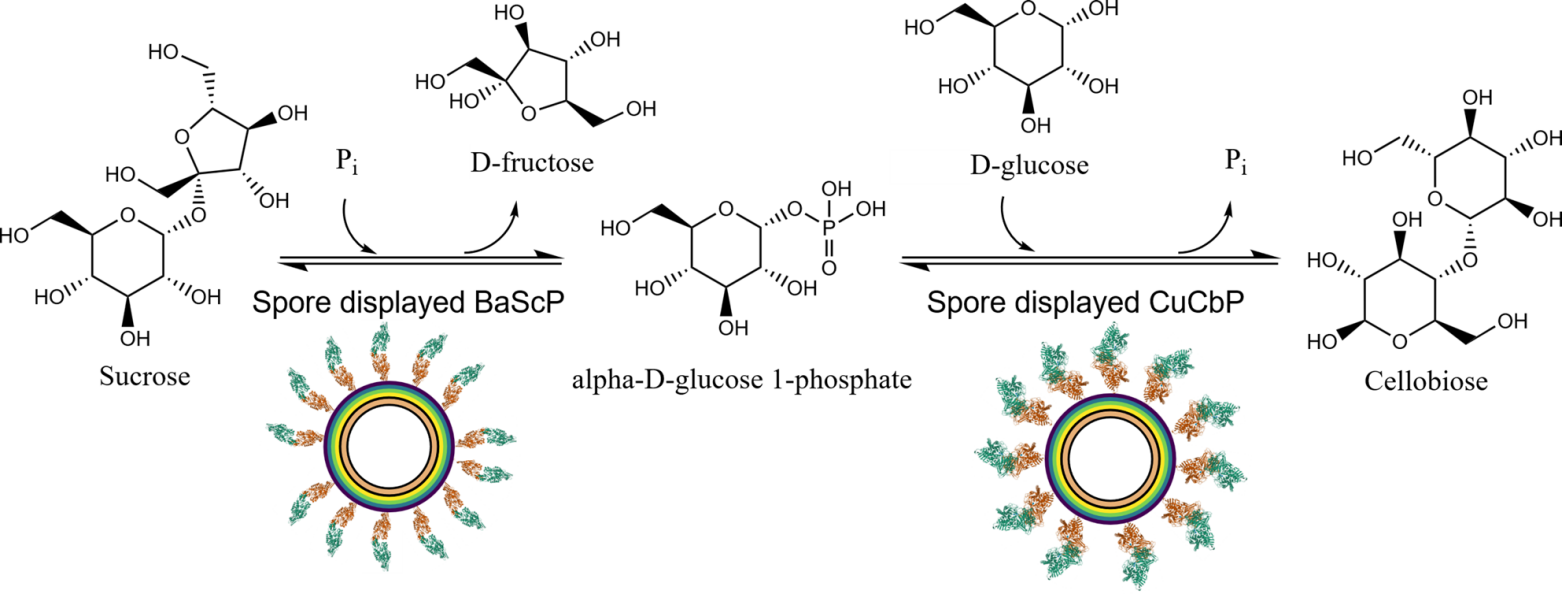
Scheme of the phosphorylase cascade for the biosynthesis of cellobiose from sucrose. BaScP, sucrose phosphorylase from *Bifidobacterium adolescentis* (EC 2.4.1.7); CbP, cellobiose phosphorylase from *Cellulumonas uda* (EC 2.4.1.20)

Besides the possibility to obtain it by acid hydrolysis of cellulose, leading to many unwanted by-products, cellobiose is typically enzymatically produced in two ways. The first possibility involves the use of cellulose in a top-down enzyme catalyzed hydrolysis. The other possibility is a bottom-up synthesis from sucrose in an enzyme cascade first shown by Taniguchi & Kitaoka et al. in 1991 [6, 7].The two step reaction converts the nonreducing disaccharide sucrose, composed of glucose and fructose, into cellobiose in a reaction sequence that mainly involves the action of two phosphorylases, offering superior control over the product profile in comparison to the processes involving cellulose [8]. Sucrose and glucose are available as bulk chemicals in high purity. In the first step, sucrose is split by sucrose phosphorylase (ScP) in the presence of phosphate, yielding α-D-glucose 1-phosphate (G1P) and fructose. In a second step, the G1P and glucose, which is added along with the sucrose, are combined into cellobiose by the action of a cellobiose phosphorylase (CbP), releasing phosphate which can be recycled (Fig. 1). As a possible addition to this cascade, a fructose isomerase can catalyze the conversion of fructose to glucose, lowering the amount of glucose needed, but also adding complexity in the form of a third enzyme [9].

Sucrose phosphorylase from the mesophilic *Bifidobacterium adolescentis* (BaScp) is the most used enzyme in the first step [9–12]. The enzyme, forming a homodimer [13], offers a comparatively high heat stability to other sucrose phosphorylases with an optimum activity reported at either 48 °C [14] or 58 °C [15]. The second enzyme, cellobiose phosphorylase is commonly a variant from *Cellulumonas uda* (CuCbP) [9, 10, 16], a monomer with around 90 kDa size [17] and has a lower optimal temperature at around 45°C [18]. The catalyzed reaction cascade is thus as a whole usually run at 45 °C, whereas a minimum of 60 °C is commonly considered to be the industrially relevant temperature, since it aids in preventing microbial growth and lowers viscosity [19].

Both phosphorylases are costly to prepare, thus cheap and easy production as well as the ability to reuse/recycle the enzymes would greatly benefit in reducing process costs [20]. In addition to lowering production costs, multiple methods have been explored to enhance the thermostability of the involved enzymes.

Enzyme immobilization is a strategy to improve heat stability and enzyme recovery after a reaction cycle. Immobilization of both enzymes can and has been carried out on solid supports, as well as in whole cell catalysts. Cerdobbel et al. used SEPA beads EC-HFA for the covalent immobilization of BaScP, also increasing the thermal stability in the process [12], while Zhong et al. co-immobilized both enzymes [11] using anion exchange resin.

However, while co-immobilization offers advantages such as substrate channeling, it is less applicable to enzymes with different operational stabilities and makes it harder to control the activity ratios of the involved enzymes [11]. In cascades involving multiple enzymes, this control is especially important for cascade flux efficiency and overall reaction yield [21]. While immobilization on solid support can offer an easy separation of the enzymes from the reaction mix through filtration or centrifugation, the immobilization process itself is often carried out as an additional step after conventional enzyme purification. This further adds to the amount of work required before the enzymes can be used in the process.

To enhance process efficiency and forgo additional immobilization steps, whole-cell biocatalysis has recently been explored for this reaction cascade. Cells of the yeast *Pichia pastoris* were used to display ScP and CbP on their surface by fusing them with cell wall proteins [20]. The cells were purified, washed and heat treated to arrest glucose uptake. Inokuma et al. showed that this enabled the immobilized enzymes to be reused for three consecutive 24 h long batch incubations, reaching yields of 83.8%, 86.5% and 81.2%.

The Gram-positive, rod-shaped bacterium *Bacillus subtilis* is a model organism in microbiology, recognized for its ability to form durable endospores. The metabolically dormant endospores, released upon lysis of the spore forming mother cell, consist of multiple proteinaceous layers and act as a survival mechanism in extreme environmental conditions such as heat and chemical reagents [22]. Many processes involving *B. subtilis* spores are generally recognized as safe (GRAS) by the FDA [23] and several products containing non-GMO *B. subtilis* spores have been approved and commercially produced for human and/or animal consumption, where they are commonly used as a probiotic [24].

Sporulation is a complex and genetically highly regulated process, occurring under unfavorable environmental conditions, e.g. nutrient starvation [25]. In a first step, the cell is asymmetrically divided into a mother cell and a forespore compartment, each containing a copy of the genome [26]. The forming spore is encapsulated in multiple layers of proteins, which are responsible for the spores resistance to oxidizing agents, lysozyme [26] and organic solvents [27]. These proteinaceous layers are the inner and outer coat, as well as the outermost layer, the crust. This crust is composed of two clusters of proteins, CotVWXYZ and CgeAB [28]. After formation of the spore is complete, the mother cell lyses, releasing the metabolically inactive spore into the environment. Their physical properties, such as size and stability enable an easy purification through multiple rounds of centrifugation and resuspension in buffer.

Thanks to the genetic amenability of *B. subtilis*, many of the proteins making up the spores coat and crust can be genetically modified to be expressed as fusion proteins linking them to a (heterologous) protein of interest [27, 29]. The spore coat was first used by Isticato et al. to display a fragment of the tetanus toxin in a proof of principle study in 2001[30]. Due to the increased stability of spores and the ease to produce them, spore displayed proteins have found various applications, including vaccine development, bioremediation and biocatalysis, making them a promising topic in biotechnological research [22, 23, 31]. Recently, spores of *Bacillus subtilis* were also used to display cellobiose dehydrogenase [32] an enzyme that oxidizes soluble cellodextrins to their corresponding lactones and finds use in biosensors [33]. Enzymes displayed on *B. subtilis* spores have so far been utilized for single-step reactions, where a substrate is converted into a product by a single enzyme. However, the synthesis of specialized sugars typically requires a multi-step process involving multiple enzymes. Chen et al. showed using spores in a two-step reaction cascade, however the two involved enzymes were produced separately and immobilized on the spore surface in a later step after spore purification [34]. To the best of our knowledge, no previous study has attempted to utilize the combined easy production, stability and reusability of spore displayed enzymes in a multi-step reaction cascade.

Here we report the immobilization of two glycosyltransferases, namely sucrose phosphorylase from *Bifidobacterium adolescentis (EC 2.4.1.7; GH13)* and cellobiose phosphorylase *Cellulumonas uda (EC 2.4.1.20; GH94)* on spores of *Bacillus subtilis* as fusion proteins with the crust protein CotY. The properties of both enzymes, which are highly relevant for the production of cellobiose were investigated with respect to their heat stability and recyclability. Both enzymes were tested in one-pot one-step reaction as well an in a two-pot reaction. Finally, we investigated the effect of precipitating excess phosphate through the addition of magnesium and calcium.

## Methods

### Transformation and strains

Replicative shuttle plasmids based on pMSE3 [35] were used to display fusion proteins on the spore surface of *B. subtilis* (modified KO7 strains) using the crust protein CotY as the fusion partner. A semi-rigid linker (EAAAK)3 links the C-terminus of CotY to either the displayed sucrose phosphorylase from *Bifidobacterium adolescentis* (strain BS02040), or the displayed cellobiose phosphorylase from *Cellulomonas uda*. Plasmid maintenance and transformation into germination deficient *Bacillus subtilis* strains was carried out as described earlier by Petersen et al.[36] (Table 1).

**Table 1:** List of relevant strains, plasmids and phenotypes in this work. The relevant insert under control of the sporulation specific P*cotYZ* promoter is given in brackets for each strain. DNA and AA sequences can be found in Sup. Data 1 and Sup. Data 2

Displayed enzyme	Strain/ plasmid	Relevant genotype
BaScP	Strain BS02040	*Plasmid* p02033: CASC1 backbone + (*cot*Y-Rigid linker-bascP) *Genomic*: Δ*cwl*D Δ*sle*B Δ*cot*Y::lox72 (germination deficient)
CuCbP	Strain BS29045	*Plasmid* p29019: CASC1 backbone + (*cot*Y-Rigid linker-cucbP) *Genomic*: Δ*cwl*D Δ*sle*B::lox72 (germination deficient)
–	Plasmid CASC1	ColE1-rep_origin (EC), KanR, Resolvase, RepE-rep_origin (BS), P*cot*YZ-promoter, (Insert)

### Sporulation

Sporulation was initiated by nutrient starvation. *B. subtilis* strains were first grown in LB with 5 g/L NaCl (LB5) media at 37 °C and 225 rounds per minute (rpm) for 14 h. For sporulation of the *B. subtilis* strains, 2x Schaeffers glucose media was used [37]. A starting optical density at 600 nm (OD_600 nm_) of 0.1 was used to inoculate the sporulation media from the LB preculture. The culture was then incubated in a shaking incubator for 48 h at 37 °C and 225 rpm.

### Spore purification

After checking samples under the microscope for successful sporulation, the culture was harvested by centrifugation (3,600 times relative centrifugal force (RCF) for 15 min). Afterwards, the pellet was resuspended in buffer (5 mM MOPS, 2.5 mM EDTA, pH 6.8) containing 0.5 mg/mL of chicken egg white lysozyme. 10 mL of this lysis solution was used per 50 mL of spore production culture. The resuspended pellet was incubated for one hour and complete lysis of the cells was verified by microscopy. The spores were then washed twice by centrifugation (2,500 RCF, 15 min) and resuspended in fresh buffer without lysozyme, followed by a final centrifugation (2,300 RCF) and resuspension in a small volume of buffer (around 2 mL/200 mL of original culture). OD_600 nm_ was then adjusted to the required optical density using buffer and spores were stored at 4 °C.

### Temperature optimum

To investigate the optimal reaction temperature of the spore immobilized enzymes, the reaction mix was incubated at temperatures between 40 °C and 60 °C degrees in increments of 5 °C. All reactions were carried out in triplicates with 150 µL reaction volume each. For the first step, BaScP displaying spores (BS02040) at an OD_600 nm_ of 1 were incubated in 20 mM MOPS buffer, adjusted to pH 6.9, containing 100 mM of sucrose and 100 mM of potassium phosphate. For step two, CuCbP displaying spores (BS29045) at an OD_600 nm_ of 1 were incubated in 20 mM MOPS buffer, adjusted to pH 6.9, containing 40 mM each of glucose 1-phosphate and glucose, as well as 100 mM of sodium chloride and 0.1 µg/mL of erythromycin. All reactions were run in a benchtop shaker (1000 rpm) at a respective temperature for 3 h. After incubation, the microcentrifuge tubes were placed in a centrifuge cooled to 4 °C and centrifuged at 10,000 RCF for 8 min. The supernatant was carefully taken off, transferred to a new microcentrifuge tube and centrifuged again. Afterwards 100 µL were transferred into a new microcentrifuge tube and freeze-dried.

### Recycling assay of individual steps

The easy separation of spores from the reaction mix by centrifugation allows for the reuse of spores for multiple reactions. To measure the loss of activity, reactions were prepared as described for the temperature optimum, with the difference of using a final spore OD_600 nm_ of 2.5 for both steps and a final reaction volume of 300 µL. After 3 h of incubation, the reaction was centrifuged for 8 min (10000 RCF). The supernatant was transferred to a new tube and centrifuged again, before 200 µL were taken off for freeze-drying. The spores were resuspended in 290 µL of fresh reaction mix, accounting for a spore pellet volume of around 10 µL in the total reaction volume of 300 µL.

### Two-pot process

When both reaction steps were carried out separately, the spores displaying sucrose phosphorylase were separated from the reaction mix after converting sucrose into G1P. The reaction product was then used together with glucose to synthesize cellobiose in a separate second step involving added spores displaying cellobiose phosphorylase.

The initial reaction was run in 20 mM MOPS buffer (pH 6.8), containing 100 mM sucrose and 100 mM phosphate. BS02040 spores (BaScP displaying) were added to a final OD_600 nm_ of 2.5. The final reaction volume of three times 2 mL was incubated for 10 h (45 °C; 1000 rpm). The reaction tubes were then centrifuged, and the supernatant transferred to new tubes, with the spore pellet being discarded. After another round of centrifugation and keeping the supernatant, the reaction was incubated at 95 °C for 5 min inactivating any remaining enzyme. After taking samples for NMR analysis, the reaction mix was pooled and pH adjusted to around 8.5 using 5 M NaOH (approximately 5 µL per mL of rection mix). The mix was then split up into seven tubes (9 tubes for the follow up experiment using triplicates at 40 mM salt concentration+controls), containing 600 µL each. To each tube, glucose was added (40 mM final), as well as BS29045 spores (CuCbP displaying; final OD 2.5), MOPS buffer at pH 8.4 (40 mM final) and NaCl (100 mM final). CaCl_2_ or MgCl_2_ were added in varying final concentrations of 40/20/10 mM (40 mM). To one tube (three tubes for the follow up experiment), ultrapure water was added instead. The volume of all tubes was adjusted to 1200 µL using ultrapure water. The reaction was run for a total of 20 h, with samples being taken for NMR analysis at various timepoints.

### Whole cascade timeline

To observe the formation of cellobiose utilizing both enzymes in a one pot reaction, BS02040 spores at a final OD_600 nm_ of 5.1 and BS29045 spores at a final OD_600 nm_ of 5.9 were suspended in a 20 mM MOPS buffer (pH 6.9) containing 100 mM of NaCl. Sucrose was added to a final concentration of 50 mM, along with glucose (40 mM) and potassium phosphate (15 mM). The final reaction volume for each triplicate was 2000 µL. The reaction was incubated at 45 °C at 1000 rpm in a benchtop thermoshaker. Samples of 150 µL were periodically taken and centrifuged in a two-stage process as described earlier, resulting in a final sample volume of 100 µL, which was freeze-dried for analysis.

### Whole cascade recycling

To monitor if the mixed spores could be used for multiple reaction cycles, whole cascade reaction mixtures were prepared as described under “Whole Cascade timeline” to a reaction volume of 500 µL. After 24 h of incubation (45 °C; 1000 rpm), the reaction was centrifuged (10000 RCF; 10 min) and the supernatant was taken off and transferred to a new microcentrifuge tube. After the supernatant was fully removed, the spores were resuspended in 480 µL of fresh reaction mix, accounting for a spore volume of around 20 µL in the total 500 µL reaction volume. The fresh reaction mix was then incubated again for a total of five cycles. During each sampling step, the removed supernatant was centrifuged once more, before a final volume of 200 µL was transferred to a new microcentrifuge tube and freeze-dried for later measurements.

### NMR spectroscopy for sample analysis

The freeze-dried reaction mixture was redissolved in D_2_O (99.9%, Sigma-Aldrich) with 0.05% 3-(trimethylsilyl)-propionic-2,2,3,3-d_4_ acid sodium salt (TSP) as an internal reference. A 1D ^1^H spectrum was run of each sample at 288 K with 32 scans. All NMR spectra were recorded on a Bruker Avance III HD 800 MHz spectrometer using a 5 mm Z-gradient CP-TCl (H/C/N) cryogenic probe. The 800 MHz spectrometer used in this study is located at the NV-NMR-Center/Norwegian University of Science and Technology (NTNU). All spectra were recorded using TopSpin 3.6 pl 7 or 4.0.8 software (Bruker BioSpin) and processed and analyzed using TopSpin 4.3.0 or 4.4.0 software (Bruker Biospin).

### Time resolved NMR spectroscopy

Time-resolved analysis was performed by preparing 900 µL of reaction mix containing 50 mM sucrose, 10 mM phosphate, 40 mM glucose and 100 mM sodium chloride in 20 mM MOPS buffer (pH 6.9). The prepared mix was then freeze-dried. Before the addition of spores, the dry reaction mix was resuspended in D_2_O to a volume of 600 µL and transferred to a 5 mm NMR tube. The sample was preheated to 45 °C in the NMR magnet, and a 1D ^1^H spectra was recorded. Then 300 µL of spores were added (BS02040 spores to a final OD_600 nm_ of 5.1 and BS29045 spores at a final OD_600 nm_ of 5.9). The sample was mixed by inverting the tube three times. The sample was put back in the magnet, and a pseudo-2D spectra was recorded by recording a 1D ^1^H spectrum every 5 min for 10 h 40 min at 45 °C. Afterwards, a ^1^-^13^ C HSQC was recorded.

### Prevention of germination and outgrowth

The *B. subtilis* strains used in this study carry deletions of the *cwlD* and *sleB* gene, which were both shown to prevent germination [38, 39]. To further inhibit outgrowth, erythromycin at a final concentration of 0.1 µg/ml was added to each reaction [40].

## Results and discussion

Sucrose phosphorylase from *Bifidobacter adolescentis* and cellobiose phosphorylase from *Cellulomonas uda* were displayed individually on spores of *Bacillus subtilis* by transforming them with a plasmid carrying a fusion protein of the spore crust protein CotY and the protein of interest. After purifying the spores, activity of the displayed enzymes was measured via a variety of assays.

### Temperature optima of both enzymes

**Fig. 2:**
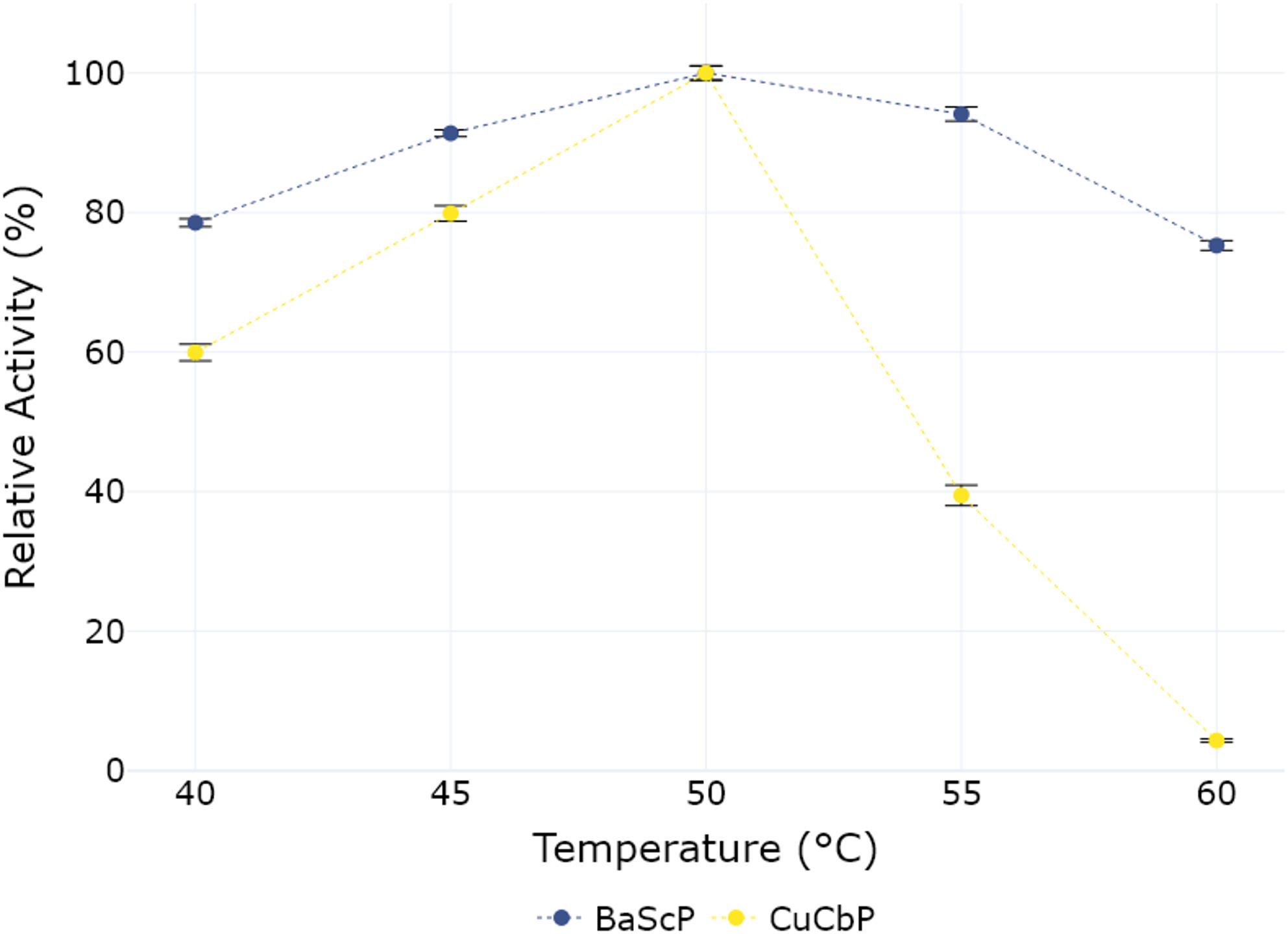
Relative activity of sucrose phosphorylase (BaScP) and cellobiose phosphorylase (CuCbP) immobilized on the spore surface of *Bacillus subtilis*. Spores at an OD_600 nm_ of 1 were incubated for 3 h with either 100 mM sucrose, phosphate and sodium chloride (BaScP) or 40 mM glucose 1-phosphate (G1P) and glucose in 20 mM MOPS buffer at pH 6.9. Relative activity was determined by expressing the conversion efficiency at each temperature as a percentage of the maximum observed conversion efficiency (50 °C), which was set to 100%. 100% relative activity translate into 26.7 mM of G1P production (BaScP)/11.2 mM cellobiose production (CuCbP). *n* = 3 for each datapoint

Multiple studies have shown that immobilization can have a positive impact on the thermostability of the displayed enzyme. This was shown for immobilization onto a spore surface for other enzymes [23, 27, 41], as well as for immobilized sucrose phosphorylase from *Bifidobacter adolescentis* in particular [15]. The activity of the immobilized enzymes was measured at 5 °C increments in the range between 40 °C and 60 °C to establish their optimal reaction temperature (Fig. 2).

For immobilized BaScP, an increase in product formation can be seen between 40 °C (21 mM) and 50 °C, reaching a maximum of 26.7 mM (100% relative activity), before dropping at higher temperatures, with 20 mM G1P formed at 60 °C. For immobilized CuCbP, a similar increase in product formation is observed between 40 °C and 60 °C, rising from 6.7 mM to 11.2 mM (100% relative activity). At higher temperatures, a sharp decline in product formation can be observed down to only 4% remaining activity at 60 °C. No activity was observed for the untransformed strain backgrounds.

For free BaScP, An Cerdobbe et al.^15^ report an optimal temperature of 58 °C, while we see a maximum conversion at 50 °C. This is closer to the optimal temperature of 48 °C which was reported by van den Broeck et al.^14^ in the initial characterization of the enzyme. The highest conversion measured for CuCbP was recorded at 50 °C, a temperature slightly higher than the optimal reaction temperature reported by Zhong et al. at 45 °C in earlier studies [18].The observed differences in optimal temperatures to previously published results might be due to differences in the reaction/measuring conditions, runtimes etc. and do not necessarily indicate an elevated temperature resistance.

To keep the reaction conditions more comparable to previously published works, it was decided to run further experiments at 45°C^10,11,18^.

### Spore immobilization offers recyclability of displayed enzymes

**Fig. 3:**
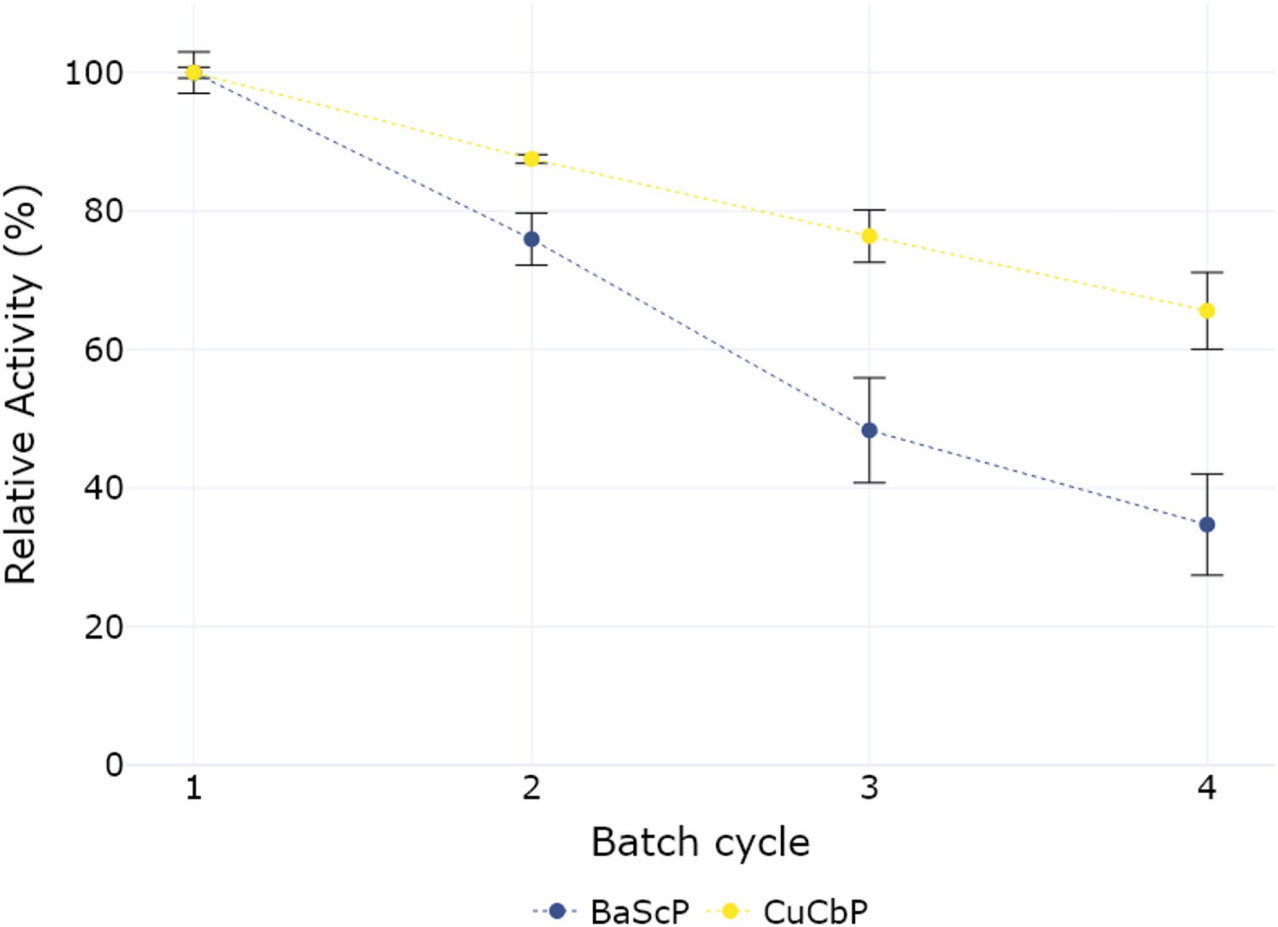
Repeated batch incubations of spores displaying one enzyme of the Cascade reaction. All reactions were incubated in 20 mM MOPS buffer (pH 6.9) at 45 °C for 3 h. Spores were separated by centrifuging twice, before being resuspended in fresh substrate. Sucrose phosphorylase (BaScP) spores were incubated with 100 mM sucrose, phosphate and sodium chloride. Cellobiose phosphorylase (CuCbP) spores were incubated with 40 mM glucose 1-phosphate (G1P) and glucose. Relative activity was determined by expressing the conversion efficiency as a percentage of the first step conversion efficiency, which was set to 100%. *n* = 3 for each datapoint

Because of their physical properties like size, spores can readily be separated from a solution by centrifugation. This opens the potential to reuse spore immobilized enzymes over multiple reaction cycles. To assess the stability of the system for the individual reaction steps, spores were recycled for 4 rounds and the final product concentration measured. Spores at an OD_600 nm_ of 2.5 were incubated for 3 h in 4 consecutive rounds (Fig. 3).

The supernatant was separated after each batch and analyzed. In the first reaction 45.2 mM of G1P was produced corresponding to a 45.2% conversion of available sucrose. After 4 rounds of recycling, the activity dropped to 35% of the initial measurement, producing 15.7 mM of glucose 1-phosphate during 3 h of incubation at 45 °C. For the second step catalyzed by CuCbP, measurements show that the spores retain 65% of their initial activity after 4 cycles, with the measured product concentration dropping from 15.6 mM in the first reaction to 10.2 mM in the fourth cycle. In the first cycle, 15.6 mM of produced cellobiose corresponds to a 39% reaction yield from available G1P. The observed differences in remaining activity might indicate a lower stability of BaScP. However, previous studies have also shown that spore displayed enzymes can detach from the spore crust during prolonged (shaking) incubation to a certain degree [36].

### Producing cellobiose in a two-pot reaction

**Fig. 4:**
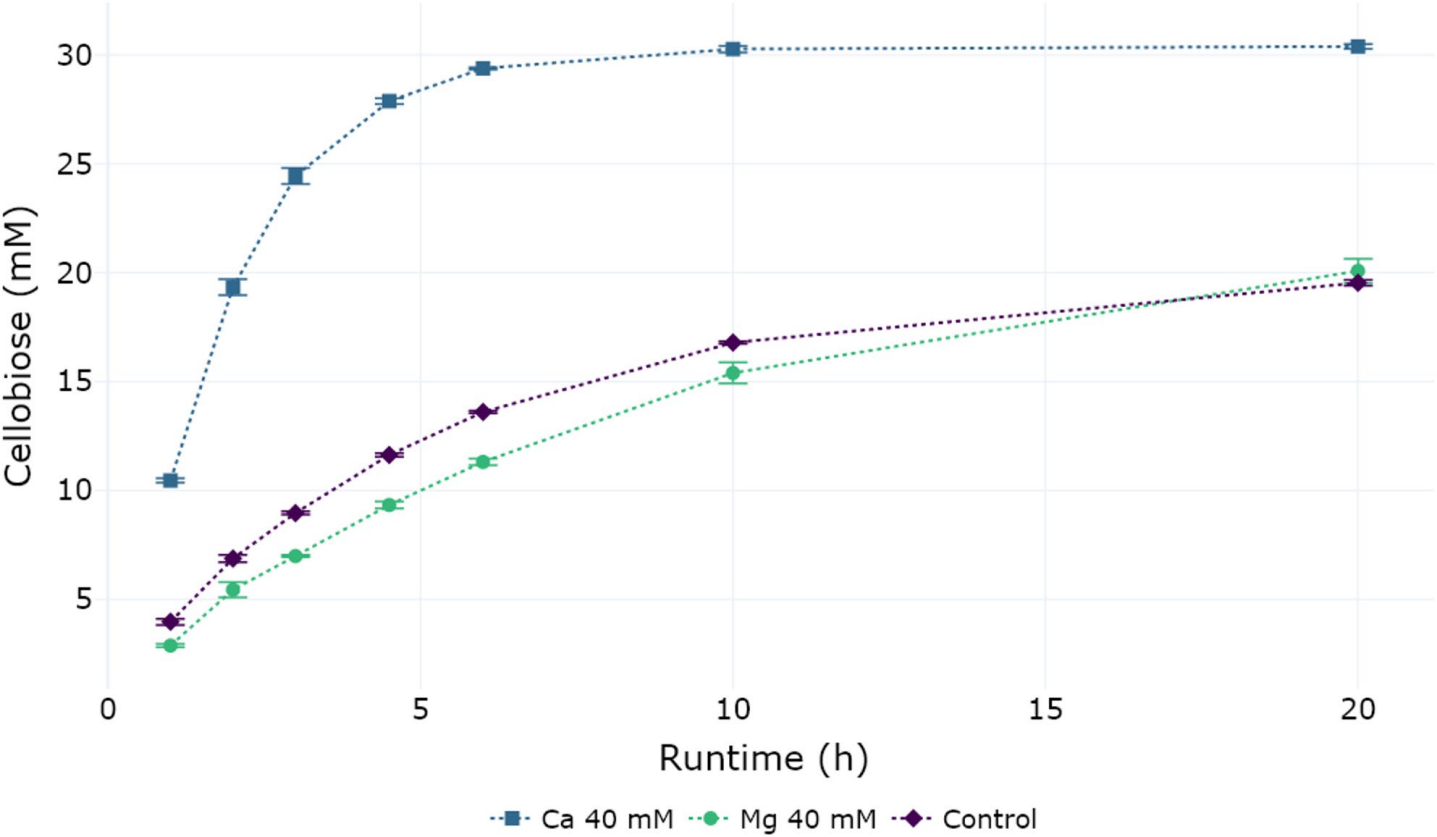
Cellobiose yield for the reaction of 38.5 mM glucose 1-phosphate and 40 mM glucose using CuCbP displayed on the surface of *Bacillus subtilis*. Calcium chloride or magnesium chloride were added to the reaction at concentrations of 40 mM in order to precipitate excess phosphate which otherwise slows down the reaction through product inhibition. The control contained no additional salt. *n* = 3 for each condition

The previously shown ability to remove spores from the reaction mix can also be used to remove them before conducting consecutive enzymatic reactions. After incubating 100 mM sucrose with phosphate and BaScp displaying spores for 10 h, the spores were removed and the concentration of G1P was determined to be 76 mM by NMR measurement. The reaction product was then used in a 1:2 dilution for the synthesis of cellobiose in the second step using CuCbP displaying spores.

When cellobiose is synthesized enzymatically, from G1P and glucose, inorganic phosphate is released. While free phosphate is a required substrate in the first step of the cascade, separation of the two steps leads to a net-consumption of phosphate in the first step and a net-release of phosphate in the second step. In the second step, free phosphate acts as an inhibitor for cellobiose phosphorylase, lowering the achievable maximum yield of the overall reaction [42]. One possibility to increase overall reaction yield is the in-situ removal of reaction products, while another is a one pot reaction, in which a small quantity of available phosphate is recycled between the first and second step [16].

Zhong et al. and others previous publications used magnesium salts to precipitate excess phosphate during cellobiose synthesis in two separate steps [18, 43]. Similar to Zhong et al., we used magnesium chloride at varying concentrations. Furthermore, the addition of calcium chloride was tested as an alternative. The K_SP_ of calcium phosphate is around five orders of magnitude higher [44, 45], resulting in a lower solubility of formed calcium phosphate salts than magnesium salts and in theory a higher removal of phosphate.

Both salts were tested at concentrations of 10, 20 and 40 mM in an initial screening experiment over the course of 20 h (*n* = 1 for each condition; Sup. Figure 4). The final yield after 20 h appeared to be concentration dependent, with higher calcium and magnesium concentrations resulting in a higher cellobiose yield. Final conversion of the initially available G1P was between 64% and 80% (25 mM to 31.3 mM) for samples with calcium addition and exhibited concentration dependence. For magnesium chloride a slight concentration dependence of the final yield after 20 h could as well be observed in comparison to the control sample, ranging from 58% (no salt) to 63% (40 mM MgCl2).

No precipitation of G1P took place upon calcium or magnesium chloride addition, which was verified by quantitative NMR analysis of the supernatant after centrifugation (Sup. Figure 1).

To confirm these findings, the experiment was repeated with freshly purified spores and calcium and magnesium concentrations of 40 mM. The first step reaction yielded 77 mM of G1P. Samples were taken at various timepoints over a duration of 20 h (Fig. 4).

While only small differences in the final yield between the control samples and samples with magnesium chloride addition were observed, the addition of 40 mM calcium to the reactions resulted in a faster progression compared to the control (2.7-fold after 3 h) and a 1.6 times higher final yield (Fig. 4). Final conversion of the initially available G1P was 79% for samples with calcium addition, 52% for magnesium added samples and 51% for control samples.

Observed discrepancies in the effect of magnesium addition compared to Zhong et al., could be due to the differences in ionic strength caused by the additional presence of 100 mM of sodium chloride, possible shifting pH during the reaction, and the lower magnesium concentration (10–40 mM) in our setup.

### One pot reaction

**Fig. 5:**
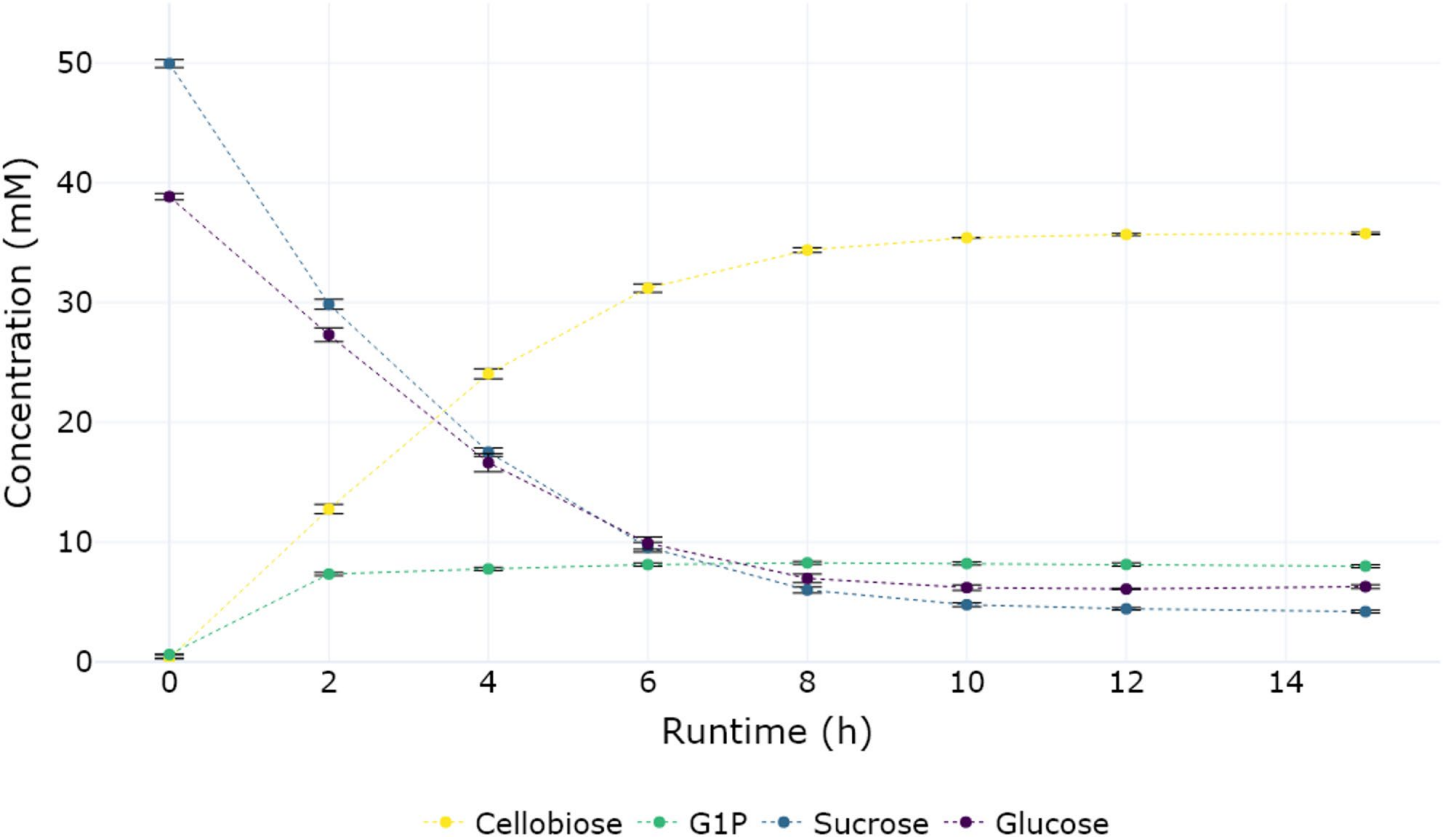
Reactant concentration over time of a one pot reaction of 50 mM sucrose, 40 mM glucose and 15 mM phosphate towards cellobiose catalyzed by BaScP and CuCbP. Spore displayed BaScp was added to an OD_600 nm_ of 5.1 and CuCbP spores to an OD_600 nm_ of 5.9. *n* = 3 for each datapoint.

To raise the effectiveness of the reaction cascade and to allow a good reaction yield without the need for salt addition and multiple steps, both steps were combined into a one-pot process.

Previous publications have already extensively covered the possible optimizations when combining both reactions into a single one-pot reaction [9, 42]. Through analysis of the previous experiments and additional data gathered (Sup. Figure 2 + 3), activities at 45 °C were estimated to be around 0.11 U/mL/OD for BaScP and 0.038 U/mL/OD for CuCbP. Earlier studies by Sigg et al. varied the ratio of both enzymes in the range 3:2–4:1 (BaScP: CuCbP) when optimizing the one-pot reaction [9]. Using OD_600 nm_ of 5.1 (BaScP) and 5.9 (CuCbP) resulted in an activity ratio of approximately 5:2.

Within the first 4 h of the reaction, the concentration of G1P rises to around 8 mM, at which it stabilizes due to the limited amount of phosphate that gets recycled between the first and second step (Fig. 5). The cellobiose concentration reaches a maximum of 36 mM after 10 h, representing a 90% yield with respect to the initially available 40 mM of glucose.

Over the course of the experiment, analysis of the mass balances revealed a higher concentration of glucose (around 2 mM after 15 h) to be present in the reaction mix then theoretically explained by the basic reaction towards cellobiose. Closer examination revealed an equal difference between theoretically remaining G1P (as a result of the phosphorolysis reaction) and actual G1P to be present in the reaction mix. Sucrose phosphorylase has in the past been shown to produce glucose as a by-product due to the double displacement mechanism of the enzyme [19]. It is thus likely that this mechanism is the reason for the observed shift in reactant concentrations. Using quantitative NMR, no significant change in overall mass balance of observed sugars was observed during the time course of the experiment (Sup. Figure 5). Petersen et al. previously demonstrated that time-resolved NMR spectra can be recorded in the presence of spore-immobilized enzymes directly within the NMR tube [36]. Building on this approach, the one-pot reaction was monitored in a time-resolved manner using pseudo-2D NMR over the course of 10 h and 40 min. Exact quantification of compound concentrations was not possible, as the suppressed water resonance at 45 °C overlapped with the product signal. Nonetheless, the time-resolved spectra revealed a stabilization of the G1P signal after approximately 80 min, accompanied by a continuous decrease in the sucrose signal as the reaction progressed (Sup. Figure 6).

### Whole cascade recycling

**Fig. 6:**
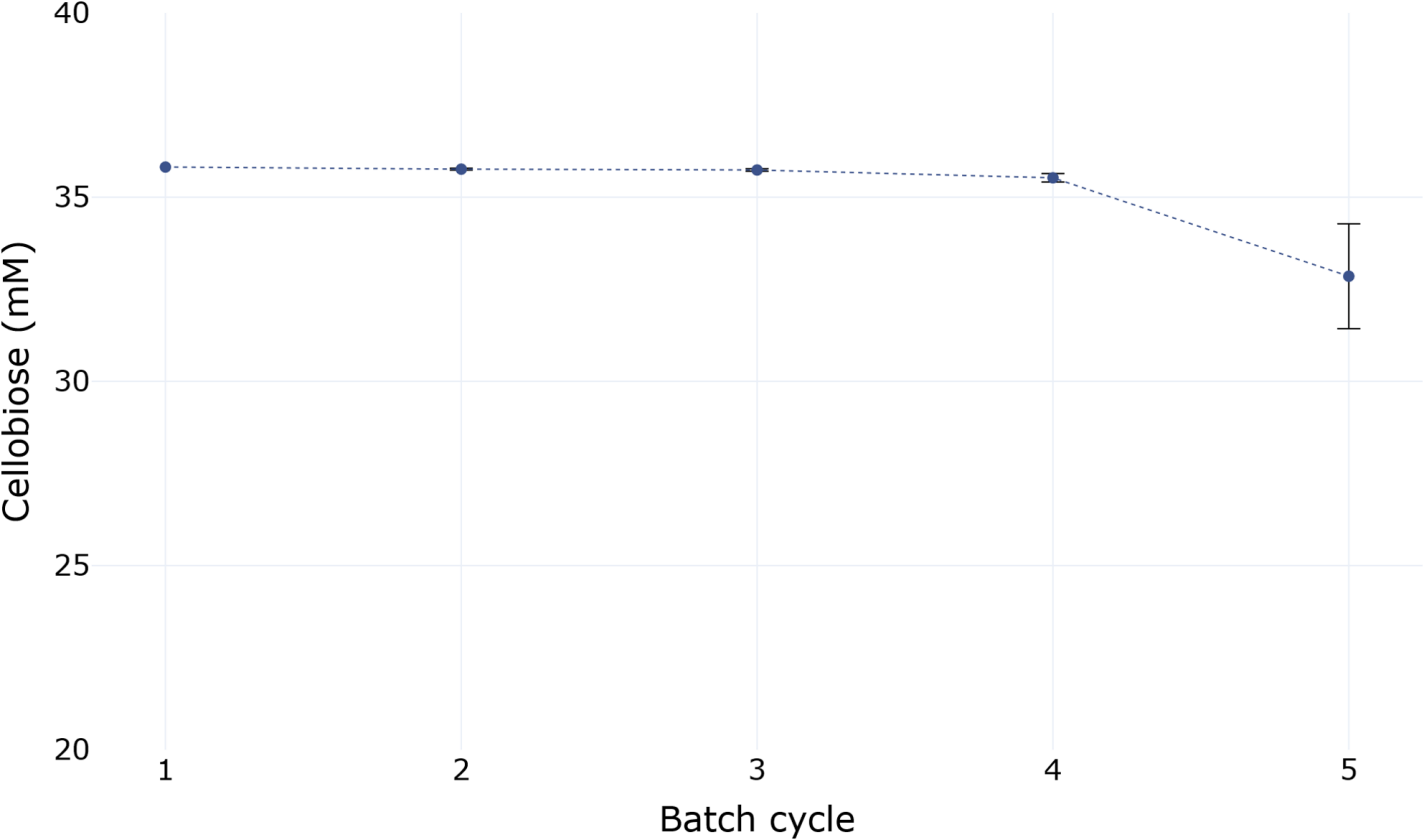
Concentration of cellobiose reached over 5 reaction cycles (24 h each). The reaction of 50mM sucrose, 40 mM glucose and 15 mM phosphate towards cellobiose was catalyzed by BaScP and CuCbP displaying spores. *n* = 3 for all datapoints, except for batch cycle 1 (*n* = 2)

To check the durability of the system we incubated the full cascade in repeated batch incubations with conditions similar to the one-pot experiment shown previously. Each reaction cycle was run for 24 h to get a better insight into the long-term stability of the spore displayed enzymes (Fig. 6).

In the four samples taken during the first 96 h, no difference in final product composition was detected with around 36 mM of cellobiose as the final yield (Fig. 6). In the fifth round of recycling, the cellobiose yield dropped to around 33 mM. We were thus able to show that not only the cascade as a whole is recyclable, but the enzymes also retain enough activity after over 96 h of incubation at 45 °C to complete a fifth reaction cycle within 24 h. While this experiment does not allow for an in-depth quantification of remaining enzyme activity, it still allows us to gain an insight into the long term stability of the system under repeated use. In the earlier time-resolved one-pot experiment, the reaction reached a steady state after around 12 h (Fig. 5). Recycling experiments for each individual type of enzyme (Fig. 3) showed a decrease in catalytic activity over repeated incubations, which is likely to also occur in recycled one-pot reactions. While BaScP exhibited the strongest decline in catalytic activity during the single enzyme recycling experiments, retaining only 35% of its initial activity after four cycles, the applied BaScP: CuCbP activity ratio of 5:2 likely compensated for this loss. The initially excess BaScP activity, together with the extended reaction time (twice that needed), enabled comparable cellobiose yields across the first four recycling cycles.

Similar to Inokuma et al., who immobilized the cascades enzymes on *Pichia pastoris*, we postulate that adapting the timeframe of each batch incubation to the time required for optimized conversion to cellobiose could improve economic feasibility [20].

## Conclusion

In this study we demonstrate that spores are a suitable immobilization platform for multistage reaction cascades. The spores displaying biocatalysts can be recovered and reused over multiple reaction cycles. In a two-pot reaction we were able to convert 76% of available sucrose into G1P before we terminated the reaction by separating BaScP displaying spores from the reaction mix and proceeded to the second step with CuCbP displaying spores. During this two-pot reaction we were able to increase the reaction yield of cellobiose from 58% (22.8 mM) to 80% (31.3 mM) through the addition of calcium resulting in the precipitation of excess phosphate ions. Both immobilized enzymes were used in a one-pot reaction, to our knowledge the first showcase of using multiple spore displayed enzymes, directly produced during sporulation, in a unified reaction setup. The simple adjustment of spore ODs has the potential to be used as an approximation for the activity of the immobilized enzymes when adjusting activity ratios in a one-pot reaction.

While in the demonstrated case the immobilization did not result in increased thermostability, multiple options have been explored in previous research to make (spore) immobilized enzymes even more fitting for application in the synthesis of cellobiose. Research has shown the potential impact different linkers of fusion proteins can have on the activity and thermostability of the fused proteins [36, 46, 47]. The enzymes involved in the reaction have also been engineered in the past to achieve a higher thermostability, making a combination of these with a variety of linkers an interesting field of research for future studies [48–50]. Co-immobilization of enzymes on the same support can have benefits such as substrate channeling [51], but the tuning of activity ratios on a single support remains a challenge in synthetic biology [10]. Immobilization of individual enzymes on the other side not only allows rapid adaption to batch-to-batch differences in activities, but could in the future also allow the use individual spore immobilized enzyme classes as building bricks in a “plug and play” composition to achieve different products and adjust reactions to changed substrates etc. Within the field of (poly)saccharide chemistry, such further additions to the reaction cascade could for example come in the form of cellodextrin phosphorylase, which extends the disaccharide cellobiose with further glucose molecules, using cellobiose/other cellodextrins and G1P as a substrate. Another possible addition is the use of fructose isomerase, to convert the fructose freed by the phosphorolysis of sucrose into glucose [7, 42].

The immobilization of glycosylic enzymes on spores enables cost-effective, scalable enzyme production on a temperature-resistant carrier that facilitates purification. The potential modularity of this approach adds to the adaptability of the system to different requirements in terms of substrate and product.

## Supplementary Information

Supplementary Material 1.

## Data Availability

Analysis of the NMR spectroscopy data is published in a Zenodo repository with the DOI: 10.5281/zenodo.17909749.
