# Supplementary material for: Spore immobilized enzymes for the multi-step synthesis of cellobiose
Source: Microb Cell Fact. 2026 Feb 3;25:43. doi: 10.1186/s12934-026-02943-w (PMC12903485; doi:10.1186/s12934-026-02943-w)
Supplement: Supplementary file 1 — Supplementary Material 1. [file 12934_2026_2943_MOESM1_ESM.docx]

# Supplementary figures and data for “Spore immobilized enzymes for multi-step synthesis of cellobiose”

All NMR analysis data can be found on Zenodo with the DOI: 10.5281/zenodo.17909749

Sup. Figure 1 Development of summed sugar integrals divided by TPS integral over time. The sugar integrals were: Glucose-1-phosphate (H1; ~5.44 ppm), α-Glucose (H1) and α-Cellobiose (reducing end)-(both ~5.23 ppm), β-Glucose (H1) and β-Cellobiose (reducing end)-(both ~4.66 ppm), as well as the β-internal peak of Cellobiose (H1; ~4.52 ppm) and Sucrose (H at the 3’C of fructose; ~4.22 ppm). Two outliers can be seen at 2 and 6 hours, due to a low sample volume because of spillage during sample preparation.

Sup. Figure 2 Activity measurements for various first step reactions in U/mL at different spores OD_600nm_.

Sup. Figure 3 Activity measurements for various second step reactions in U/mL at different spores OD_600nm_.


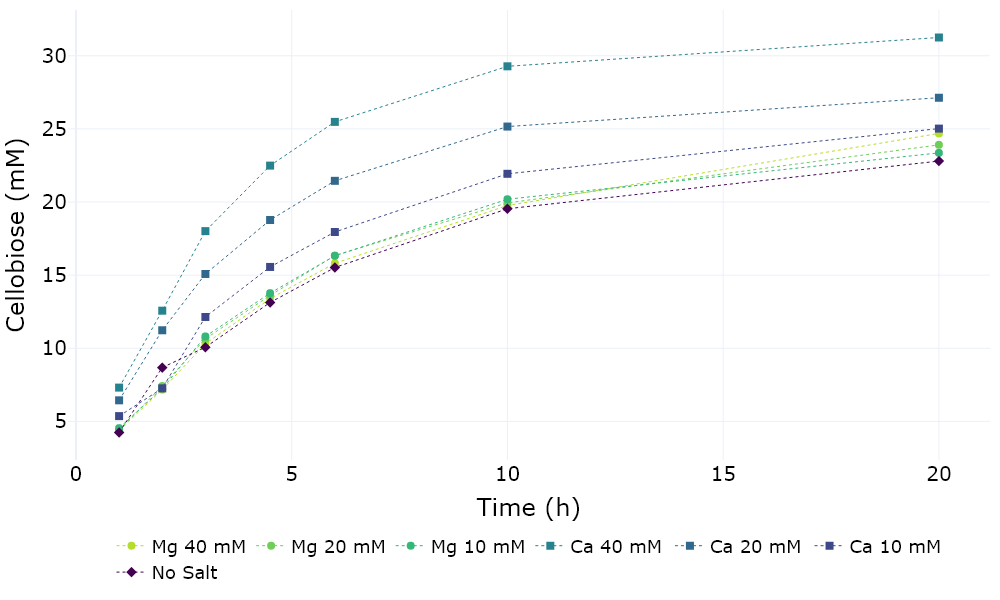


*Sup. Figure 4 Cellobiose yield for the reaction of 38 mM glucose 1-phosphate and 40 mM glucose using CuCbP displayed on the surface of Bacillus subtilis. Different salts were added to concentrations between 10-40 mM in order to precipitate excess phosphate which otherwise slows down the reaction through product inhibition. While only small differences in the final yield between the control samples and samples with magnesium chloride addition were observed, the addition of 40 mM calcium to the reactions resulted in a faster progression (almost 2-fold after 3 hours) and a 1.4 times higher final yield (Figure 4). Final conversion of the initially available G1P was between 64% and 80% (25 mM to 31.3 mM) for samples with calcium addition and was concentration dependent. For magnesium chloride a slight concentration dependent difference in final yield after 20 hours was also observed in comparison to the control sample, ranging from 58% (no salt control) to 63% (40 mM MgCl2*). n=1 for each condition

Sup. Figure 5 Development of summed sugar integrals divided by TPS integral over time. The sugar integrals were: Glucose-1-phosphate (H1; ~5.44 ppm), α-Glucose (H1) and α-Cellobiose (reducing end)-(both ~5.23 ppm), β-Glucose (H1) and β-Cellobiose (reducing end)-(both ~4.66 ppm), as well as the β-internal peak of Cellobiose (H1; ~4.52 ppm) and Sucrose (H at the 3’C of fructose; ~4.22 ppm). Measurements were performed in triplicates. Average and standard deviation are shown.


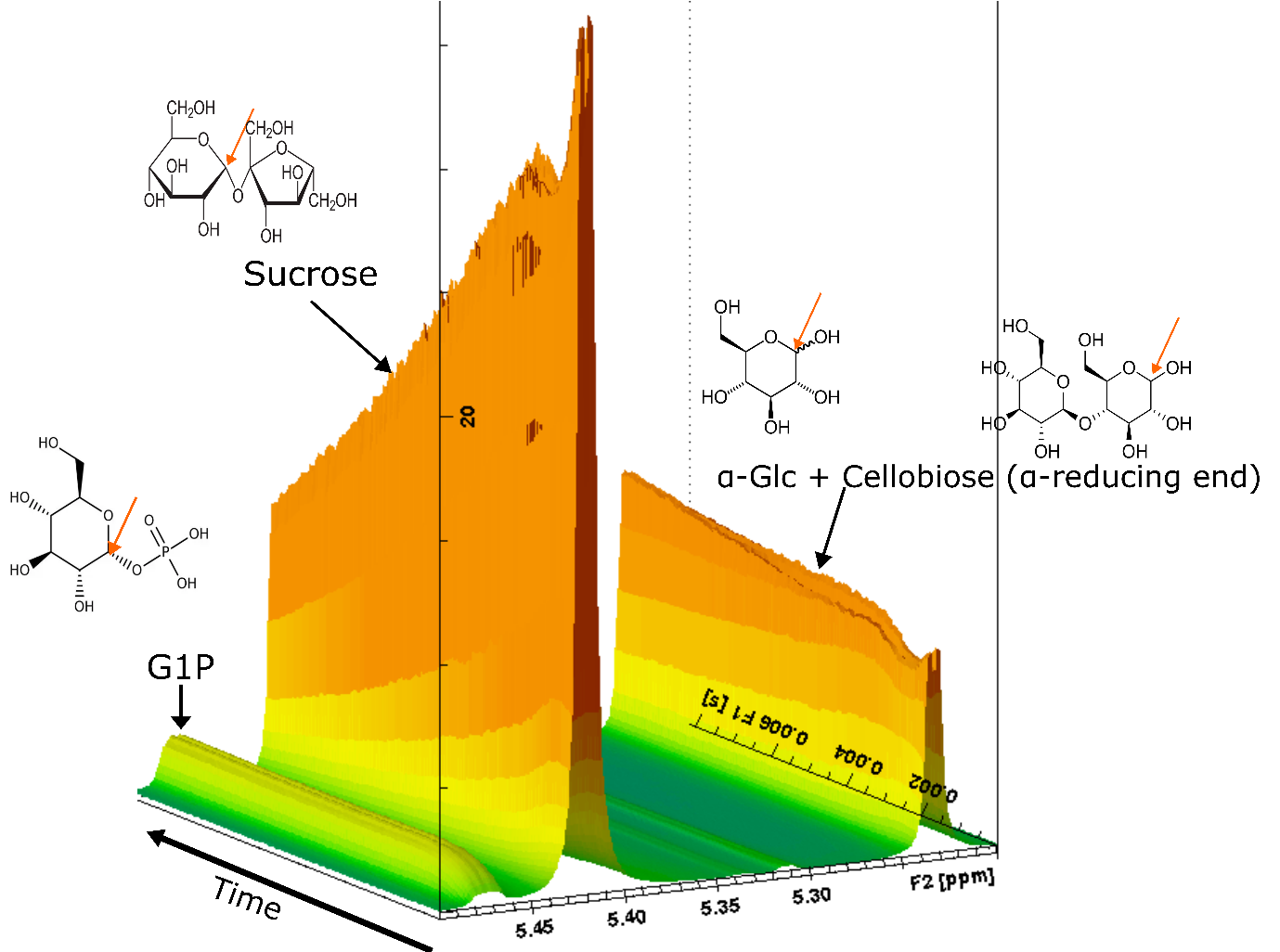


Sup. Figure 6 Recorded pseudo-2D NMR over the course of 10 hours and 40 minutes of a one pot reaction.

Sup. Figure 7 Integrated peaks over time for the recorded pseudo-2D NMR shown in Sup. Figure 6.

## Supplementary data

### Sup. data 1 DNA&AA sequence of CotY-BaScP fusion protein

LOCUS p02033_extraction 2046 bp DNA linear

DEFINITION SucP_Bifido in pCASCADEv1.0.

FEATURES Location/Qualifiers

gene 1..>486

/label="spore coat protein Y CDS"

linker 487..534

/label="linker"

gene 535..2046

/label="Sucrose phosphorylase Bifidobacterium adolescentis"

ORIGIN

1 atgagctgcg gaaaaaccca tggccggcat gagaactgtg tatgcgatgc agtggaaaag

61 attttagcag agcaggaggc agttgaagaa cagtgtccga ctggctgcta taccaacctt

121 ttaaacccta cgattgctgg aaaagacaca attccgtttc tcgtttttga taaaaaaggc

181 ggattgttct ccacattcgg aaacgtaggg ggatttgtgg atgatatgca atgctttgaa

241 tccattttct tccgcgtcga aaaattatgc gattgctgtg caacactgtc tattttacgc

301 ccggtcgatg tcaaaggcga taccttaagt gtttgccacc cttgcgaccc ggatttcttc

361 gggctagaaa aaacagattt ctgcattgaa gtggatctcg gatgcttctg cgcgattcag

421 tgcctgtcac cagagctagt tgacagaaca tcgcctcaca aagataaaaa gcatcatcac

481 aatggagagg cggcagcaaa agaagccgcc gcgaaggaag cggcagctaa atcaatgaaa

541 aacaaagttc agctgattac atacgccgac cggcttggcg atgggacaat taaatcgatg

601 acagatattc ttcggacgcg cttcgacgga gtttacgacg gagtgcacat ccttccgttc

661 tttacacctt ttgacggtgc tgacgccggg tttgatccaa ttgatcacac gaaagtggat

721 gagcgactgg gctcttggga tgatgtcgcc gaactgtcca aaacgcataa tattatggtg

781 gacgccatcg ttaaccatat gtcttgggaa agtaaacaat ttcaggatgt gttggcgaaa

841 ggggaggaaa gcgaatacta tccgatgttt ttgacaatgt caagtgtatt tccgaatggg

901 gcaacggaag aagaccttgc aggaatctac agaccgcggc cgggactgcc gtttactcac

961 tataagtttg ctggcaaaac cagacttgtt tgggtaagct ttacaccgca gcaggtcgat

1021 atcgatactg atagcgacaa aggatgggag tacttaatgt ccatttttga tcagatggcg

1081 gcgtcacacg tcagttacat ccgacttgac gcagtcggtt acggtgccaa agaggctggc

1141 acaagctgct ttatgacacc aaaaacgttt aagttgatct ctcgtctgcg agaagaaggc

1201 gtcaagcgag gcttggaaat tctgattgag gtacactcat attacaaaaa acaggttgaa

1261 attgccagca aagtcgacag ggtttacgat tttgcgcttc cacctctcct cctccacgcc

1321 ttatcaacag gacacgtgga accagtagcc cattggacgg atattagacc gaataatgct

1381 gttactgtcc tggataccca cgatggcata ggggttattg acatcggctc tgatcaactt

1441 gaccgttcac ttaaaggcct tgtgccggac gaagatgttg acaatcttgt aaatacaatt

1501 catgccaata cacacggaga atctgaggcc gcgactgggg cagcggcaag taacttggac

1561 ctgtatcaag ttaactctac ctactactcc gcgttaggat gcaatgatca acactacatt

1621 gcggcaagag cggtccagtt tttcctgcct ggggtccctc aagtctacta cgttggagca

1681 ctggcaggca agaatgatat ggaattgttg aataaaacta ataatggcag agatataaat

1741 cggcactatt actcgacggc tgaaatcgac gagaacttaa aacggccggt cgttaaagcc

1801 cttaatgcat tagcaaaatt tagaaatgag ctcgacgcat ttgacgggac attctcttac

1861 acgacaccga cagacacttc tatttccttc acttggagag gggaaacatc ggaagcaacc

1921 ttaacttttg aaccaaaacg cggcttggga gtcgataata cgactccagt tgcaatgctg

1981 gaatggcatg atagcgcggg tgaccacaga tctgatgatc tcattgcgaa tccgccggtt

2041 gtggcg

//

LOCUS p02033_extraction_translation 682 aa linear

DEFINITION SucP_Bifido in pCASCADEv1.0.

FEATURES Location/Qualifiers

CDS 1..>162

/label="spore coat protein Y CDS"

linker 163..178

/label="linker"

gene 179..682

/label="Sucrose phosphorylase"

ORIGIN

1 mscgkthgrh encvcdavek ilaeqeavee qcptgcytnl lnptiagkdt ipflvfdkkg

61 glfstfgnvg gfvddmqcfe siffrveklc dccatlsilr pvdvkgdtls vchpcdpdff

121 glektdfcie vdlgcfcaiq clspelvdrt sphkdkkhhh ngeaaakeaa akeaaaksmk

181 nkvqlityad rlgdgtiksm tdilrtrfdg vydgvhilpf ftpfdgadag fdpidhtkvd

241 erlgswddva elskthnimv daivnhmswe skqfqdvlak geeseyypmf ltmssvfpng

301 ateedlagiy rprpglpfth ykfagktrlv wvsftpqqvd idtdsdkgwe ylmsifdqma

361 ashvsyirld avgygakeag tscfmtpktf klisrlreeg vkrgleilie vhsyykkqve

421 iaskvdrvyd falpplllha lstghvepva hwtdirpnna vtvldthdgi gvidigsdql

481 drslkglvpd edvdnlvnti hanthgesea atgaaasnld lyqvnstyys algcndqhyi

541 aaravqfflp gvpqvyyvga lagkndmell nktnngrdin rhyystaeid enlkrpvvka

601 lnalakfrne ldafdgtfsy ttptdtsisf twrgetseat ltfepkrglg vdnttpvaml

661 ewhdsagdhr sddlianppv va

//

### Sup. data 2 DNA&AA sequence of CotY-CuCbP fusion protein

LOCUS CuCbP_in_pCASCADEv1.0_extraction 3000 bp DNA linear

DEFINITION CuCbP in pCASCADEv1.0

FEATURES Location/Qualifiers

CDS 1..>486

/label="spore coat protein Y CDS"

linker 487..534

/label="semi rigid Linker"

gene 535..3000

/label="cellobiose phosphorylase"

CDS 535..3000

/label="CuCbP"

ORIGIN

1 atgagctgcg gaaaaaccca tggccggcat gagaactgtg tatgcgatgc agtggaaaag

61 attttagcag agcaggaggc agttgaagaa cagtgtccga ctggctgcta taccaacctt

121 ttaaacccta cgattgctgg aaaagacaca attccgtttc tcgtttttga taaaaaaggc

181 ggattgttct ccacattcgg aaacgtaggg ggatttgtgg atgatatgca atgctttgaa

241 tccattttct tccgcgtcga aaaattatgc gattgctgtg caacactgtc tattttacgc

301 ccggtcgatg tcaaaggcga taccttaagt gtttgccacc cttgcgaccc ggatttcttc

361 gggctagaaa aaacagattt ctgcattgaa gtggatctcg gatgcttctg cgcgattcag

421 tgcctgtcac cagagctagt tgacagaaca tcgcctcaca aagataaaaa gcatcatcac

481 aatggagaag ccgctgcgaa agaagccgcc gcaaaagaag ctgcagccaa atcaatgcgg

541 tacggacact tcgatgacga agcgagagaa tatgtgatta cgacaccaca cacaccttac

601 ccatggataa attacttagg aagcgaacag ttctttagtc ttttatctca ccaagctggt

661 gggtactcat tctatcggga tgcgaagatg agacgcttaa ccaggtatcg gtataacaac

721 ataccagcgg atgcaggtgg ccgctatctg tacgtcaatg acggcgggga cgtgtggacc

781 ccatcgtggc tgcctgttaa agctgatctt gaccactttg aggcgagaca cggattgggg

841 tactcaacca ttactggtga aagaaacgga gtgcgcgttg aaacattatt cttcgttcca

901 gtgggtgaga acgctgaggt ccaaaaggtc acagtgacta acaccagcga ctcgtacaag

961 tcgctgacct tattttcctt tgtggagttc tgcttatgga acgctcaaga cgaccagacg

1021 aattaccaga ggaatctttc tataggagag gtagaggtcg agcaggagag tccccacgga

1081 tcggcaatct atcatcggac ggagtaccgt gaacggcggg atcattacgc cgttttcgct

1141 gtaaacaccc aggctgaggg attcgatacc gaccgcgaca cattcgtggg agcttacaac

1201 agcctgggcg aggctgctgt tccgcttaaa ggggagtcag caaactccgt tgcctcaggt

1261 tggtacccta tcggttcaca cagcgttgct gtaagtttgg cgccgggaga gtcaagggag

1321 ttagtctatg ttttgggtta tgtagaaaat cctgacgaag agaaatgggc ggacgacgcg

1381 aagcaggtcg ttaataagga acgcgctcat gccctgctgt caagatttgc tacaagcgag

1441 cagactgacg ccgctttcgc ggccctgaaa gactattgga ctgacttact ctccacctac

1501 tctgtgtcga gtaacgacga gaaactggac agaatggtga atatctggaa tcaataccag

1561 tgtatggtga cattcaacat gagcagatca gccagtttct tcgagacagg tattgggcgt

1621 ggtatgggat tcagagattc taatcaggat ctcctgggct tcgtacatct gattcctgaa

1681 agagctcgag aaagaatcat tgacatcgcg agcacccagt tcgctgacgg gagcgcgtac

1741 caccagtatc agccgctgac aaagcgtgga aataatgata tcggttcggg gtttaacgat

1801 gatcctttat ggctcattgc tggaactgcc gcctatatca aggaaaccgg tgatttcagc

1861 atcttagacg aaccggtccc attcgacaat gaaccaggca gcgaagttcc tcttttcgag

1921 cacttgaccc ggagctttga atttaccgtc acccatcgcg ggcctcacgg gttgccatta

1981 atcggacgcg ctgattggaa cgactgtctt aatttgaact gtttctctac aacacctggt

2041 gagtcttttc agactacaga gaaccaggcg ggcggcgtcg cggaatcaac cttcatcgca

2101 gcccagttcg tgttatatgg cgagcagtac gcagagttag ctgcacgccg cggattagca

2161 gatgtggcgg acagagcgag aggacacgtt gccgagatgc gggacgctct tctcaccgac

2221 gggtgggatg gcagctggtt cctgcgggcc tatgactact acggtaaccc aattggtacc

2281 gatgcccacg atgaaggtaa aatatggatc gaaccacaag ggttcgcggt gatggctgga

2341 gttggggtgg gcgaaggacc acaggacacc gacgcacccg ccatcaaggc gttagacagt

2401 gtgaacgaga tgctggcgac tgaccacggc atggttcttc agtaccctgc ctacacaacc

2461 taccaagttc acatgggaga ggtaagcacc tacccgcctg gctacaagga gaatggtggc

2521 atattttgtc ataacaatcc ctgggttatc attgctgaga ctgtagtagg acgaggtggc

2581 cgtgctttcg actactataa gagaatcacc ccagcctacc gtgaagacat aagcgacgtc

2641 caccgcctgg aaccatacgt ctacgcgcaa atgatagccg gcaaagaggc tgttcgtcac

2701 ggagaagcca agaatagctg gctgacggga acagctgcat ggaatttcgt aaccgtgagc

2761 caatacctgc tcggagttcg tccggagtac gacggcttag tggttgatcc acagattggg

2821 ccggacgtcc ccagtttcac agtcacgcgg gtggctcgcg gcgccacgta tgagattacg

2881 gtcactaatt cgggtacgga cggatcgaga gggcgtttgg tcgtcgatgg aacacccgta

2941 gagggcaatc ttgttcccta cgctccggct ggtagcacag ttcgagttga tgtgacgttg

//

LOCUS CuCbP_in_pCASCADEv1.0_extraction_translation 1000 aa linear

DEFINITION CuCbP in pCASCADEv1.0

FEATURES Location/Qualifiers

CDS 1..>162

/label="spore coat protein Y CDS"

CDS 163..178

/label="semi rigid Linker"

gene 179..1000

/label="cellobiose phosphorylase"

CDS 179..1000

/label="CuCbP"

ORIGIN

1 mscgkthgrh encvcdavek ilaeqeavee qcptgcytnl lnptiagkdt ipflvfdkkg

61 glfstfgnvg gfvddmqcfe siffrveklc dccatlsilr pvdvkgdtls vchpcdpdff

121 glektdfcie vdlgcfcaiq clspelvdrt sphkdkkhhh ngeaaakeaa akeaaaksmr

181 yghfddeare yvittphtpy pwinylgseq ffsllshqag gysfyrdakm rrltryrynn

241 ipadaggryl yvndggdvwt pswlpvkadl dhfearhglg ystitgerng vrvetlffvp

301 vgenaevqkv tvtntsdsyk sltlfsfvef clwnaqddqt nyqrnlsige veveqesphg

361 saiyhrteyr errdhyavfa vntqaegfdt drdtfvgayn slgeaavplk gesansvasg

421 wypigshsva vslapgesre lvyvlgyven pdeekwadda kqvvnkerah allsrfatse

481 qtdaafaalk dywtdllsty svssndekld rmvniwnqyq cmvtfnmsrs asffetgigr

541 gmgfrdsnqd llgfvhlipe rareriidia stqfadgsay hqyqpltkrg nndigsgfnd

601 dplwliagta ayiketgdfs ildepvpfdn epgsevplfe hltrsfeftv thrgphglpl

661 igradwndcl nlncfsttpg esfqttenqa ggvaestfia aqfvlygeqy aelaarrgla

721 dvadrarghv aemrdalltd gwdgswflra ydyygnpigt dahdegkiwi epqgfavmag

781 vgvgegpqdt dapaikalds vnemlatdhg mvlqypaytt yqvhmgevst yppgykengg

841 ifchnnpwvi iaetvvgrgg rafdyykrit payredisdv hrlepyvyaq miagkeavrh

901 geaknswltg taawnfvtvs qyllgvrpey dglvvdpqig pdvpsftvtr vargatyeit

961 vtnsgtdgsr grlvvdgtpv egnlvpyapa gstvrvdvtl

//
